# A Guide to the Short, Long and Circular RNAs in Hypertension and Cardiovascular Disease

**DOI:** 10.3390/ijms21103666

**Published:** 2020-05-22

**Authors:** Priscilla R. Prestes, Michelle C. Maier, Bradley A. Woods, Fadi J. Charchar

**Affiliations:** School of Health and Life Sciences, Federation University Australia, Ballarat, VIC 3350, Australia; p.prestes@federation.edu.au (P.R.P.); m.maier@federation.edu.au (M.C.M.); b.woods@federation.edu.au (B.A.W.)

**Keywords:** long non-coding RNA, circular RNA, microRNA, hypertension, coronary artery disease, atherosclerosis, cardiovascular disease, heart disease

## Abstract

Cardiovascular disease (CVD) is the leading cause of morbidity and mortality in adults in developed countries. CVD encompasses many diseased states, including hypertension, coronary artery disease and atherosclerosis. Studies in animal models and human studies have elucidated the contribution of many genetic factors, including non-coding RNAs. Non-coding RNAs are RNAs not translated into protein, involved in gene expression regulation post-transcriptionally and implicated in CVD. Of these, circular RNAs (circRNAs) and microRNAs are relevant. CircRNAs are created by the back-splicing of pre-messenger RNA and have been underexplored as contributors to CVD. These circRNAs may also act as biomarkers of human disease, as they can be extracted from whole blood, plasma, saliva and seminal fluid. CircRNAs have recently been implicated in various disease processes, including hypertension and other cardiovascular disease. This review article will explore the promising and emerging roles of circRNAs as potential biomarkers and therapeutic targets in CVD, in particular hypertension.

## 1. Hypertension and Cardiovascular Disease (CVD)

Hypertension (HT) is a major cause of death in adults in developed countries and leads to many other comorbidities. Both genetic and environmental factors contribute to the development of the disease, although their relative contributions are still poorly defined. Importantly, undiagnosed, uncontrolled or untreated hypertension is a risk factor for the development of other cardiovascular diseases, including coronary artery disease (CAD), stroke, heart failure (HF), atrial fibrillation etc. [1].

The sympathetic nervous system, endothelial homeostasis and the renin-angiotensin-aldosterone system (RAAS) are involved in the maintenance of normal blood pressure (BP), and the instability of these mechanisms are believed to be involved in the development of HT [2].

The causes of HT are still poorly understood. However, genetic factors play a pivotal role in the development of HT. Genetic variations interact with several environmental factors that increase BP and progress to HT development. These factors include obesity, insulin resistance, high alcohol intake, high salt intake (in salt-sensitive patients), aging, sedentary lifestyle, stress, low potassium and calcium intake [3].

According to public health records, both the cost and number of hypertensive adults has been predicted to increase in the future [4,5].

In this review article, we will provide an overview of the contribution of non-coding RNAs (ncRNA) in the development of many cardiovascular disease (CVD), mainly focusing on circular RNAs (circRNA) and hypertension.

## 2. Hypertension Genetics

The heritability of HT is estimated to be around 30% [6]. Genome wide association studies have greatly expanded our understanding of the genetic contribution of mutation to HT. A recent study of more than one million participants with European ancestry identified 535 novel BP related loci [7]. Several loci identified have been functionally implicated in the transforming growth factor beta (TGFβ) pathway. This pathway regulates sodium homeostasis in the kidney and is thought to affect ventricular remodelling [8]. Furthermore, elevated TGFβ serum levels have been associated with hypertension development [9]. Early research into RAAS has identified polymorphisms in the angiotensinogen, angiotensin converting enzyme, angiotensin II type 1 and renin genes, with links to their plasma levels contributing to an increase in BP [10]. Another study, by Zeller et al. [11], described that the messenger RNA (mRNA) levels of eight candidate genes (CRIP1, MYADM, TIPARP, TSC22D3, CEBPA, F12, LMNA, and TPPP3) accounted for up to 13% of changes in BP. Interestingly, CRIP1 was also strongly associated with cardiac hypertrophy. Finally, epigenetic modifications exploring ncRNA, such as microRNAs (miRNA), long non-coding RNAs (lncRNA) and circRNAs, have been described in HT and CVD. Furthermore, it is also important to highlight the relevance of epigenetic reprogramming during cardiac differentiation, development and remodelling. These characteristics and their implications will be discussed in the next sections [12,13,14,15].

## 3. Non-Coding RNAs (ncRNA)

**Figure 1:**
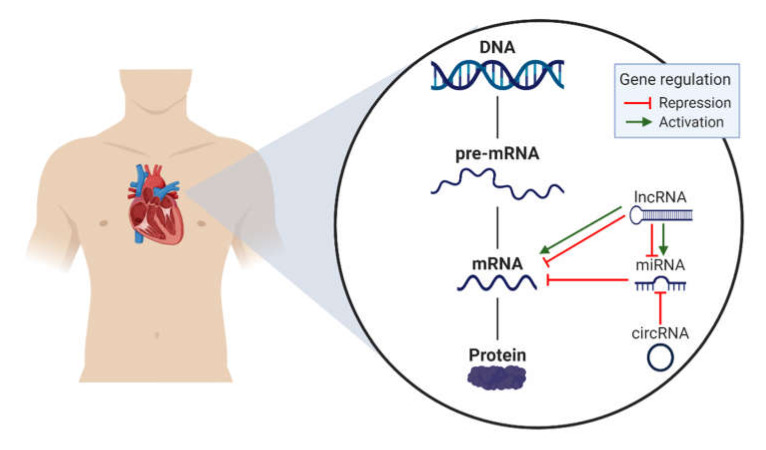
Non-coding RNA regulation. Legend: mRNA, messenger RNA; lncRNA, long non-coding RNA; miRNA, microRNA; circRNA, circular RNA.

NcRNAs are RNA types transcribed from DNA but not translated into protein, generated by a specific alternative splicing and generally regulating gene expression transcriptionally, post-transcriptionally and translationally (Figure 1) [16,17,18]. ncRNAs include microRNA (miRNA), long non-coding RNA (lncRNA) and circRNA, which have been found to play a role in the development and progression of CVD [19].

### 3.1. MicroRNAs in CVD and Hypertension

**Figure 2:**
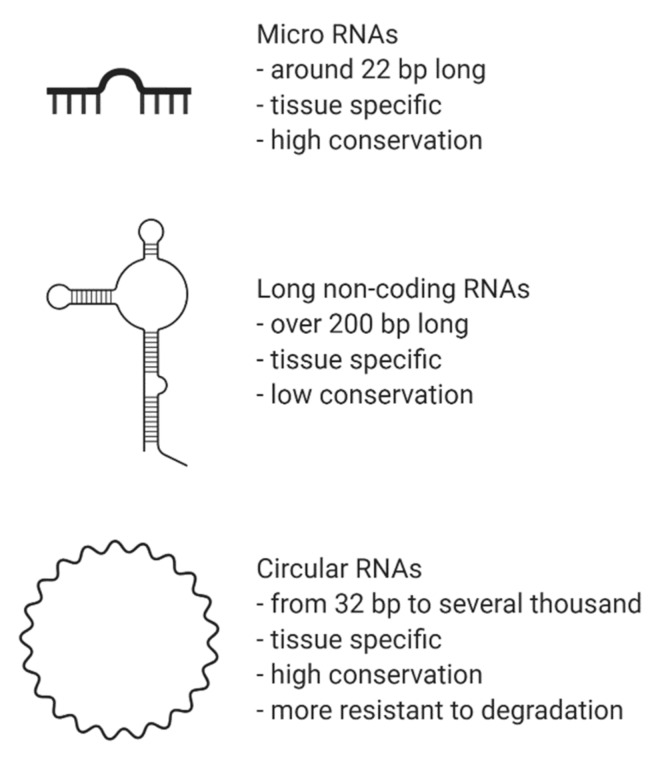
Brief characteristics of non-coding RNAs. Legend: bp, base pairs.

MiRNAs are non-coding RNAs endogenous, highly conserved, single-stranded, small (~22 nucleotides), stable and ubiquitously detectable, including in the circulation of an organism, both in plasma and serum (Figure 2) [20]. MiRNAs regulate gene expression post-transcriptionally by binding to the 3’-untranslated regions of target mRNAs [21]. Each miRNA potentially targets many unique mRNAs, inhibiting translation and/or inducing degradation of the target mRNA depending on the number and accessibility of binding sites. Higher complementarity between the miRNA and its target(s) and greater levels of mRNA inhibition or degradation are observed [16]. Changes in miRNA expression levels have been associated with several CVDs including hypertension, atherosclerosis, CAD, myocardial infarction (MI), HF and cardiac arrhythmias, suggesting their potential use as therapeutic targets and diagnostic and prognostic biomarkers [22]. Several review articles have highlighted the main findings on miRNA expression and associations with CVDs [15,23,24,25]. In this review, we will provide a few examples to illustrate their importance.

Recent studies have described approximately 50 miRNAs associated with essential hypertension and over 30 with HF and MI, with many of those miRNAs described as promising biomarkers [23,25]. The renin-angiotensin system is a balanced network that regulates blood pressure with multiple miRNAs involved. The downregulation of miR-34b, miR-361-5p, miR-362-5p, and miR-181a and the upregulation of miR-34c-5p, miR-449b, miR-571, miR-765, miR-483-3p, miR-143/145, miR-126, miR-196a, miR-132, miR-212, miR-451, and miR-21, independently or as a group, impact this system and seem to cause an increase in blood pressure [25]. MiR-21 is expressed in many cell types related to cardiovascular health, including vascular smooth muscle cells (VSMC), vascular endothelial cells, myocardial cells, cardiac fibroblasts and blood [26]. Its expression is closely related to the development and progression of HT and related to target organ damage, including the regulation of the renin angiotensin system, inflammatory cytokines and endothelial function [27]. In addition to the miR-21 role in HT development, research into cardiac dysfunction post MI suggests its critical involvement in cardiac fibroblast activation and cardiac fibrosis via the TGFβ/Smad7 signalling pathway [28].

Elevated plasma levels of miR-1, miR-133a, miR-499 and miR-208a have been described in acute MI, highlighting its potential use as a biomarker for early diagnosis [29]. In addition, miR-19a/19b are upregulated in heart failure patients after MI and the delivery if its mimics to the heart increases cardiac proliferation and regeneration, indicating a possible compensatory mechanism in response to stress [30].

Higher levels of miRNA-29a are strongly correlated with hypertrophic cardiomyopathy and fibrosis of the heart [31]. MiRNAs have also been described in the progression and regression of atherosclerosis, via the regulation of lipoprotein homeostasis. The downregulation of miR-145 encourages plaque formation in the vasculature of patients through decreased VSMC differentiation [32]. One of the first studies to use miRNA for potential medical intervention in atherosclerosis, treated ApoE−/− mice, which are pre-disposed to developing atherosclerosis, with a lentivirus, aiming to increase the expression of miR-145. The upregulation of miR-145 after lentivirus treatment resulted in a decrease in plaque formation in the vasculature of the ApoE−/− mice [33].

Another role for miRNAs is their potential involvement in the regulation of angiotensin-converting enzyme 2 (ACE2). ACE2 inactivates the BP regulating angiotensin II (Ang II) by cleaving it to the Ang 1–7 products [34,35]. Reduced expression levels of ACE2 have been reported in hypertension [36]. More intriguing is that it also acts as the tissue receptor for coronavirus (COVID-19) [35,37,38]. A study by Liu et al. found that miR-200c-3p directly targets the 3′-untranslated region of ACE2 and downregulates its protein expression [39]. These observations have interesting implications for the development of novel miRNA therapeutics for both hypertension and COVID-19.

Importantly, recent studies also highlight the relevance of miRNAs in modulating the levels of ACE2 in other CVD. MiRNA let-7b is upregulated in hypoxic pulmonary hypertension which decreases levels of ACE2. The in vitro decrease of let-7b eased the development of hypoxic pulmonary hypertension [40]. Patients with chronic kidney disease and on haemodialysis have high levels of serum miR-421 and low levels of Ang 1–7 products and ACE2, which have been described as being able to contribute to the development of atherosclerosis [41]. Lastly, miR-125b is a negative regulator of ACE2 after high glucose treatment. The downregulation of ACE2 was hindered after knocking down miR-125b in human kidney cells, which also reduced reactive oxygen species and apoptosis after high-glucose treatment. The interactions between miR125b and ACE2 present a potential therapeutic target in diabetic nephropathy [42].

Despite ongoing investigations into miRNAs and CVD, its incorporation into clinical practice has not occurred. This is mainly occurring due to the lack of an easy-to-handle, fast, reliable, and inexpensive method to determine miRNAs levels. However, many miRNAs are currently being used as part of clinical trials, which may lead to early diagnosis, prevention and treatment.

### 3.2. Long Non-Coding RNAs and Their Involvement in CVD and Hypertension

LncRNAs are greater than 200 nucleotides in length, tissue specific and poorly conserved across species. They play a role in transcriptional and post-transcriptional gene regulation and mRNA translation, being involved in epigenetic modifications, the modulation of alternative splicing and transcription, or interacting with mRNAs and proteins in the cytoplasm to regulate gene expression (Figure 2) [25].

Similarly to miRNAs, a large number of lncRNAs has been described to be involved in the critical regulation of several cardiac disorders, highlighting their role in the development and progression of CVD [25]. Importantly, expression of genes such as ACE2 are not only regulated by miRNAs, but also lncRNAs. ALT1 is a lncRNA downregulated in hypoxia induced, growth arrested human umbilical vein endothelial cells and a direct target of ACE2 [43].

LncRNA growth arrest-specific 5 (GAS5) was shown to regulate VSMC and endothelial cells (EC) in animal models [44]. Vascular remodelling is strongly correlated with the dysfunction of VSMC and EC, suggesting a role of GAS5 to the development of essential hypertension [44]. Another study, using microarray analysis, identified 145 differentially expressed lncRNAs in the ipsilateral renal cortex when comparing spontaneously hypertensive and normotensive rats [45].

A cluster of lncRNAs from the Myh7 gene was identified as functionally significant in cardiac hypertrophy in mice [46]. Myh7, a myosin heavy chain associated RNA transcript (Mhrt) was irreversibly reduced after the induction of cardiac hypertrophy through transverse aortic surgery (TAC). The overexpression of Mhrt reduced cardiac hypertrophy and fibrosis leading to an improvement in cardiac function was observed when compared to TAC operated mice without reactivated Mhrt [46].

The lncRNA cardiac hypertrophy associated transcript, or CHAST, is upregulated in cardiomyocytes of TAC operated mice and involved in cardiomyocyte hypertrophy. CHAST is also upregulated in patients with cardiac hypertrophy and cardiomyocytes derived from human embryonic stem cells. Research shows that the overexpression of CHAST induces cardiomyocyte hypertrophy in cell and animal models. Meanwhile, its silencing prevents TAC induced cardiac remodelling with no toxicological side effects. Interestingly, pleckstrin, the opposite strand of CHAST, inhibits cardiomyocyte autophagy and hypertrophy, suggesting that CHAST could be a potential target to prevent cardiac remodelling, while indicating a general role of lncRNAs in heart diseases [47].

The lncRNA myocardial infarction associated transcript (MIAT) has been identified as promoting cardiac fibrosis and remodelling after MI [48]. Cardiac fibrosis presents after MI and cardiac remodelling of the infarct region is key in sustaining myocardial integrity, through preventing wall rupture during the healing process. Conversely, fibrosis caused by cardiac remodelling increases cardiac stiffness and impairs cardiac function that can lead to HF [49]. Furthermore, other studies revealed that MIAT acts as pro-hypertrophic in cardiomyocytes through sponging anti-hypertrophic miR-93 and miR-150 [50,51]. The oxygen deficiency caused by MI leads to a significant loss in viable cardiomyocytes by necrotic cell death and apoptosis. The lncRNA, mitochondrial dynamic related lncRNA (Mdrl) and cardiac apoptosis-related lncRNA (Carl) are both reduced after MI [52,53]. Increased expression of Mdrl and Carl inhibited cardiomyocyte apoptosis through reduction in miRNAs miR-361 and miR-539, resulting in reduced infarct sizes. All these studies suggest that lncRNAs are critical regulators of cardiac fibrosis and cardiomyocyte survival in hypertrophic and infarcted hearts by gene regulation interference, through interactions with other ncRNAs such as miRNAs.

A study by Wang et al. [54] identified the lncRNA cardiac hypertrophy associated epigenetic regulator (Chaer) as essential for the development of cardiac hypertrophy in a mouse model of pressure overload induced failing hearts. Interestingly, Chaer knockdown significantly supressed chemically induced hypertrophy by phenylephrine, but did not interfere with myocyte morphology at basal level. Chaer directly interacts with polycomb repressor complex 2 (PRC2), which inhibits downstream genes involved in cardiac hypertrophy. The interaction between Chaer and PRC2 is briefly induced post hormone or stress stimulation and this interaction is a requirement for the epigenetic reprogramming that activates genes involved in cardiac hypertrophy. The inhibition of Chaer expression in the heart prior to the onset of pressure overload decreases cardiac hypertrophy and dysfunction, but is not impacted in post-stressed hearts [54].

### 3.3. Circular RNAs: What Are They and How Do They Function?

**Figure 3:**
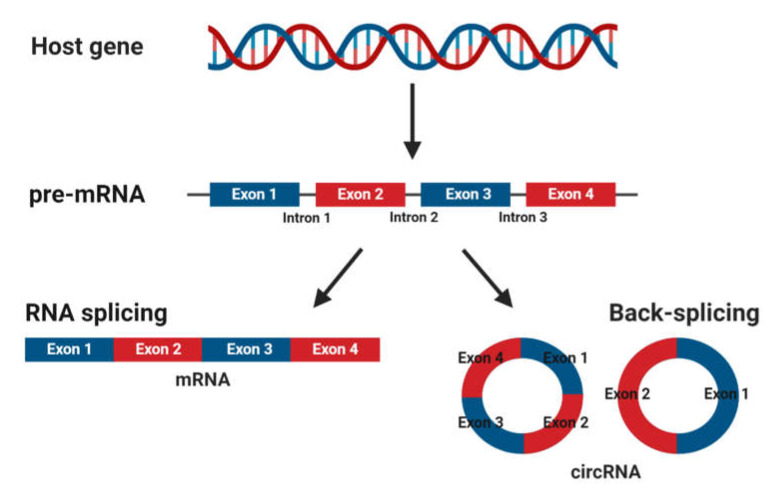
Biogenesis of mature messenger RNA (mRNA) and circular RNA (circRNA) from precursor mRNA (pre-mRNA). Pre-mRNA undergoes RNA splicing to form a mature mRNA or back-splicing to form circRNAs.

CircRNAs are abundant, underexplored ncRNAs. Recent studies revealed that large numbers of circRNAs are endogenous, highly conserved and stable in mammalian cells and prevalent in disease states (Figure 2) [7]. Although mRNA and circRNAs both originate from precursor-mRNAs, they are formed differently, giving them unique characteristics. mRNAs are formed by RNA splicing where introns are removed, and certain exons are included or excluded to create alternative coding mature mRNAs. This process creates linear mRNAs with exposed poly(A) tails. This characteristic leaves them prone to degradation by RNases [55]. Meanwhile, circRNAs are formed by back-splicing, promoting the circularization process where exons and/or introns converge onto each other, potentially protecting them from degradation and conferring a half-life of approximately 48 h, around five times more stable than linear mRNAs (Figure 3) [56].

**Figure 4:**
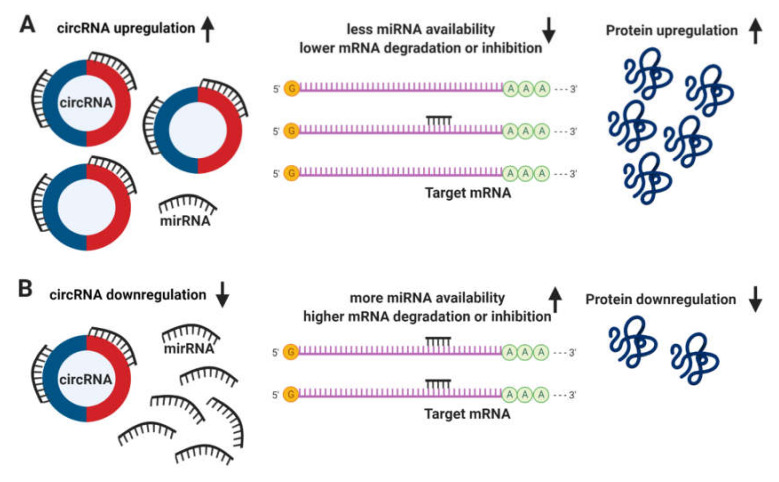
Circular RNA (circRNA) functions. (**A**) The upregulation of circRNAs leads to a decrease in microRNA (miRNA) availability, messenger RNA (mRNA) degradation or inhibition, resulting in an increase of protein translation. (**B**) Conversely, the downregulation of circRNAs leads to more miRNA available and higher levels of mRNA degradation or inhibition, leading to a downregulation in protein translation.

The definitive function of circRNAs still remains unclear. It has been proposed that circRNAs regulate the expression of linear mRNA transcripts both directly, via the competition with the splicing machinery [57]; and indirectly, acting as sponges to miRNAs due to the presence of multiple binding sites, allowing them to interact with and sequester cellular miRNAs preventing the performance of their roles on post-transcriptional regulation (Figure 4) [58,59].

Importantly, circRNAs make up 1% of total RNA being expressed widely in various cell types and may have a regulatory function in human disease, with a pivotal role in the initiation and progression of various types of biological processes [58], potentially acting as a biomarker for the discovery and investigation on the progression of disease. However promising, research to identify and characterize circRNAs has mostly been performed using bioinformatics and in silico approaches and a limited number of studies have investigated their function in situ or in vivo to establish their involvement in disease.

The properties described above and promising research in the field of cancer genomics using circRNAs as biomarkers is encouraging and should be explored and translated into cardiovascular genomics research. This is extremely relevant, as conventional methods for controlling risk factors and initiating early treatment in CVD intervention have often led to poor prognosis. Current biomarkers usually detect the disease at later stages of development, increasing the need for the discovery of new biomarkers for prevention or at early onset of disease. This further highlights the benefits of using circRNAs as potential biomarkers in CVD (Table 1).

Emerging evidence of circular RNAs in cardiovascular disorders has demonstrated differential expression in both healthy and diseased hearts [60,61,62,63]. However, the relevance of circular RNAs to the cardiovascular system remains poorly characterised, and an improvement in understanding of circRNA involvement in CVD will form a basis for the development of these RNAs as biomarkers for discovery, prediction and therapeutic agents. Importantly, the combination of genetic sequencing and bioinformatics discovery has enabled the identification of many novel circRNAs.

### 3.4. Circular RNAs and Their Involvement in CVD

#### 3.4.1. Circular RNAs and Hypertension

Limited studies have explored the association between circRNAs and hypertension development. Wu et al. [64] used microarrays to study circRNAs in blood samples of 54 hypertensive patients and 54 healthy controls and found 13 downregulated and 46 upregulated circRNAs. Validation of these findings in a larger cohort identified that hsa_circ_0005870 was significantly downregulated in the blood of hypertensive patients. CircRNA-miRNA networking predicted binding sites between hsa_circ_0005870 and several miRNAs, including hsa-miR-619-5p. Interestingly, miR-619-5p had the largest number of in silico predicted mRNA targets. The Kyoto Encyclopedia of Genes and Genomes (KEGG) pathway analysis of this interaction proposed that the TGFβ signalling pathway may play a pathological role in hypertension [64].

Additional case-control studies identified two unique circRNAs, hsa_circ_0037909 and hsa_circ_0037911, strongly associated with the hypertensive phenotype with BP greater than 140/90mmHg [65]. CircRNA-miRNA networking identified a potential interaction with miR-637, which has been proposed to decrease C-reactive protein levels, by activating inflammatory pathways in hypertensive patients [86]. Further analyses have also identified a positive relationship between hsa_circ_0037911 and serum creatinine. However, a potential mechanism for the increase of serum creatinine and C-reactive protein or a possible interplay between the circRNA-miRNA interactions and this change have not been identified [66].

Lastly, two unique circRNAs, hsa_circ_0126991 and hsa_circ_0014243, were upregulated in hypertensive patients and were predicted to bind to miR-10a-5p [65,66]. This miRNA is downregulated in hypertension, CAD and the dysfunction of the vascular endothelium which contributes to hypertension development [87]. A major caveat of all the above studies has been the small number of subjects studied and further studies in larger cohorts are warranted.

#### 3.4.2. Circular RNAs, Myocardial Infarction and Heart Failure

A microarray study identified 29 upregulated and 34 downregulated circRNAs in a mouse model of post-MI leading to HF and predicted numerous circRNA-miRNA interactions. This was one of the earliest studies into circRNAs in MI, improving our knowledge into the potential dysregulation of circRNAs and CVDs [88].

CircRNA Cdr1as is upregulated in mice with MI induced injuries and hypoxia treated cardiomyocytes. Its overexpression aggravated the infarct size in vivo and led to cell apoptosis in mouse cardiomyocytes. Importantly, Cdr1as acts as a sponge for miR-7a and impacts its downstream targets. The upregulation of miR-7a had previously been described as protective during MI injury [69]. Therefore, decreasing expression levels of Cdr1as may increase levels of miR-7a and this could act as a new therapeutic strategy for the treatment of MI.

Interestingly, circTtc3 has also been shown to be upregulated in myocardium and an in vitro model of hypoxia in cardiomyocytes after MI [70]. The overexpression of circTtc3 decreased hypoxia induced ATP depletion, which in turn, was increased following circTtc3 downregulation in neonatal rat ventricular myocytes [70]. The knock down of circTtc3 also affected cardiac dysfunction after MI, increasing apoptosis in cardiomyocytes under cardiac ischemia and dysfunction [70].

circNfix is overexpressed in adult hearts of humans, rats and mice. However, its expression seems to be mediated by a transcription factor bound to a super enhancer. Knocking down circNfix promoted an increase in cardiomyocyte proliferation and angiogenesis, which prevented apoptosis post MI, decreased cardiac dysfunction and improved prognosis after MI [72].

Conversely, circFndc3b is downregulated in mouse hearts post MI and in human cardiac tissue of ischemic cardiomyopathy patients. Its overexpression decreased apoptosis in cardiomyocytes, improved vascularisation and left ventricular function [73].

These examples highlight the possible involvement of circRNAs in cardiac repair, function and remodelling after MI, providing a novel therapeutic target for its treatment and prognosis. However, cardiac circRNAs are yet to be described in blood upon MI and the measurement of cardiac troponin levels continues to be the gold standard biomarker for MI [89].

The potential value of circRNAs as a biomarker has been demonstrated in whole blood samples of patients who suffered MI. It has been demonstrated that patients with lower blood levels of MICRA, or myocardial infarction associated circular RNA, are at a greater risk of developing HF following acute MI [71]. This circRNA is downregulated in the blood of MI patients when compared to healthy controls and was predicted to be a strong indicator of left ventricular dysfunction [90]. This highlights the benefits of MICRA as a prognostic tool to evaluate HF risks.

#### 3.4.3. Circular RNAs, Atherosclerosis and CAD

The Chr9p21 region is a well-established risk locus for atherosclerosis. Antisense noncoding RNA in the INK4 locus (ANRIL) located in this region is associated with atherosclerosis, regulating molecular pathways and cellular functions [91,92]. Studies show that individuals with a high circANRIL-ANRIL ratio exhibit no signs of atherosclerosis [93].

Recent studies have demonstrated that the upregulation of certain circRNAs sponges’ miRNAs impact the proliferation and invasion of cells involved in CAD. circRNA-0044073 targets miR-107 [74]; while circCHFR targets mir-370 [77], circRNA-0003575 targets miR-148a-3p [78].

Interestingly, circRNA hsa_circ_0003575 is upregulated in damaged endothelium, a crucial part in the development of atherosclerosis and related to post-oxidised low-density lipoprotein treatment [94]. The knockdown of this circRNA decreases apoptosis in human umbilical vein endothelia cell (HUVEC) [94].

Importantly, the use of RNA sequencing identified thousands of circRNAs in CAD. Pan et al. [95] described 1259 annotated and 381 novel circRNAs in atherosclerotic lesions. The combination of those results and histology examination identified 54 circRNAs upregulated and 12 downregulated, suggesting a possible involvement in the CAD pathology. Another study, by Yu et al. [96], identified 2283 downregulated and 85 upregulated in CAD patients. The top 100 differentially regulated circRNA originated from genes related to metabolism and protein modification. Furthermore, the expression profiles of six circRNAs were validated between atherosclerotic coronary arteries and peripheral blood mononuclear cells, warranting further investigation into their use as potential biomarkers. Hsa_circ_0001879, hsa_circ_0004104 and hsa_circ_0001445 have been suggested as novel biomarkers for CAD after experimental validation showed an upregulation in patients when compared to controls [79,97].

#### 3.4.4. Circular RNAs and Cardiomyopathy

The titin gene, responsible for coding proteins that make muscle cells (including cardiac muscle), undergoes complex alternative splicing, including back splicing, creating around 80 circRNAs. Some of these circRNAs are differentially expressed in dilated cardiomyopathy, but not in hypertrophic cardiomyopathy [98]. Another study using deep sequencing identified 82 novel circRNAs, the authors validated differential expression of circATXN10, circCHD7, circDNAJC6 and circSLC8A1 in biopsies from dilated cardiomyopathy patients, in comparison to controls [99]. Other studies found that circSLC8A1 is also upregulated in heart tissue of patients with dilated cardiomyopathy and hypertrophy induced by pressure overload [85,100].

Other circRNAs were also found to be upregulated in physiologically induced hypertrophy. The upregulation of heart-related circRNA (HRCR) suppresses miR-223, overexpressing the gene for activity-regulated cytoskeleton-associated protein (ARC) and reducing the levels of cardiac hypertrophy induced by isoproterenol [82]. In contrast, circRNA-000203 was upregulated in angiotensin-II infused mice, which served as and endogenous sponge for miR-26b-5p and miR-140-3p that target Gata4 levels, leading to an increase in cardiac hypertrophy [84].

Finally, an investigation into human induced pluripotent stem cells (hiPSCs) and hiPSC-derived cardiomyocytes (hiPSC-CMs) identified 5602 circRNAs illustrating the array of possibilities to be explored in this field. Interestingly, the investigation of circRNA expression during cardiac differentiation and human heart specific enrichment in foetal tissues found circSLC8A1, circCACNA1D, circSPHKAP and circALPK2 to be differentially expressed [100].

Strong evidence suggests that circSLC8A1 may also be a promising candidate as a biomarker of cardiomyopathy or a possible therapy target warranting further investigation [85].

## 4. Future Directions

Whilst the role of long non-coding RNAs and miRNAs is better established, the role of circRNAs in CVD and HT is still in its infancy. The examples provided in this review suggest a relevant role for these circRNAs in the development, heart physiology and pathogenesis of the many facets of heart disease. However, many more studies are still needed to define their role and implications in CVD. The continuous research into this area will provide clues towards understanding how circRNAs are involved mechanistically in normal cardiac function and the effects caused by their dysregulation. A better understanding of how circRNAs affect gene expression and their specificity needs to be determined, either if targeted directly or mediated by miRNAs. If it is true that these circRNAs target many miRNAs, their effect could be very non-specific. We believe that there is a need to understand how these molecules target specific genes or miRNAs. The first question that remains open is, where do circRNAs fit in the molecular pathway of cardiovascular development and disease? An understanding of that question will clarify their clinical relevance and provide insights into their potential use in disease prevention and prediction and therapeutic targets or prognostic tools.

## Figures and Tables

**Table 1:** Summary of circRNAs implicated in cardiovascular diseases, including species and sample types studied and differential expression direction.

Disease	Circular RNA	Species	Sample	Expression	References
Hypertension	hsa_circ_0005870	Human	Blood	Down	[64]
	hsa_circ_0037909	Human	Blood	Up	[65]
	hsa_circ_0037911	Human	Blood	Up	[66]
	hsa_circ_0126991	Human	Blood	Up	[67]
	hsa_circ_0014243	Human	Blood	Up	[68]
Myocardial infarction	mmu_circ_0001878	Mouse	Cardiomyocytes	Up	[69]
	circTtc3	Rats	Cardiomyocytes	Up	[70]
	MICRA	Human	Blood	Down	[71]
	circNfix	Mouse	Cardiomyocytes	Up	[72]
	circFndc3b	Mouse Human	Cardiomyocytes	Down	[73]
Atherosclerosis	circRNA-0044073	Human	Blood	Up	[74]
	ocu-ciR-novel-18038	Rabbit	Blood	Down	[75]
	ocu-ciR-novel-18298	Rabbit	Blood	Up	
	ocu-ciR-novel-15993	Rabbit	Blood	Up	
	ocu-ciR-novel-17934	Rabbit	Blood	Down	
	ocu-ciR-novel-17879	Rabbit	Blood	Up	
	ocu-ciR-novel-18036	Rabbit	Blood	Up	
	ocu-ciR-novel-14389	Rabbit	Blood	Up	
	circANRIL	Human	Blood	Up	[76]
	circCHFR	Human	VSMC	Up	[77]
	circRNA-0003575	Mouse	Endothelial cells	Up	[78]
Coronary artery disease	hsa_circ_0001879	Human	Blood	Up	[79]
	hsa_circ_0004104	Human	Blood	Up	
	hsa_circ_0124644	Human	Blood	Up	[80]
	hsa_circ_0098964	Human	Blood	Up	
	hsa_circ_0030769	Human	Blood	Up	[81]
	hsa_circ_0079828	Human	Blood	Up	
	hsa_circ_15486-161	Human	Blood	Up	
	hsa_circ_0122274	Human	Blood	Up	
	hsa_circ_16316-13	Human	Blood	Up	
	hsa_circ_0140538	Human	Blood	Up	
Cardiomyopathy	mmu_circ_0000254	Mouse	Cardiomyocytes	Down	[82]
	hsa_circ_0076631	Human	Cardiomyocytes and Serum	Up	[83]
	circRNA_000203	Mouse	Ventricular Cardiomyocytes	Up	[84]
	circSlc8a1	Mouse	Cardiomyocytes	Up	[85]

Legend: ciR, circular; circ, circular; circANRIL, circular antisense non-coding RNA in the INK4 locus; circCHFR, circular checkpoint with fork-head associated and ring finger; circFndc3b, circular fibronectin type III domain containing 3B; circNfix, circular nuclear factor I X; circSlc8a1, circular solute carrier family 8 member a1; circTtc3, circular tetratricopeptide repeat domain 3; hsa, Homo sapiens; MICRA, myocardial infarction-associated circular RNA; mmu, Mus musculus; ocu, Oryctolagus cuniculus; VSMC, vascular smooth muscle cells.

## Abbreviations

ACE2	angiotensin-converting enzyme 2
Ang II	angiotensin II
ANRIL	antisense noncoding RNA in the INK4 locus
ARC	activity-regulated cytoskeleton-associated protein
BP	blood pressure
CAD	coronary artery disease
Carl	cardiac apoptosis-related lncRNA
Chaer	cardiac hypertrophy associated epigenetic regulator
CHAST	cardiac hypertrophy associated transcript
circRNA	circular RNA
COVID-19	coronavirus
CVD	cardiovascular disease
EC	endothelial cells
GAS5	growth arrest-specific 5
HF	heart failure
hiPSC	human induced pluripotent stem cells
hiPSC-CM	hiPSC-derived cardiomyocytes
HRCR	heart-related circRNA
HT	hypertension
KEGG	Kyoto Encyclopedia of Genes and Genomes
lncRNA	long non-coding RNA
Mdrl	mitochondrial dynamic related lncRNA
Mhrt	myosin heavy chain associated RNA transcript
MI	myocardial infarction
miRNA	microRNA
mRNA	messenger RNA
ncRNA	non-coding RNA
PRC2	polycomb repressor complex 2
RAAS	renin-angiotensin-aldosterone system
TAC	transverse aortic surgery
TGFβ	transforming growth factor beta
VSMC	vascular smooth muscle cells
